# Short-term effects of implemented high intensity shoulder elevation during computer work

**DOI:** 10.1186/1471-2474-10-101

**Published:** 2009-08-10

**Authors:** Mette K Larsen, Afshin Samani, Pascal Madeleine, Henrik B Olsen, Karen Søgaard, Andreas Holtermann

**Affiliations:** 1 National Research Centre for the Working Environment, Copenhagen, Denmark; 2 Laboratory for Ergonomics and Work-related Disorders, Center for Sensory-Motor Interaction (SMI), Department of Health Science and Technology, Aalborg University, Aalborg, Denmark; 3 Institute of Sports Science and Clinical Biomechanics, University of Southern Denmark, Odense, Denmark

## Abstract

**Background:**

Work-site strength training sessions are shown effective to prevent and reduce neck-shoulder pain in computer workers, but difficult to integrate in normal working routines. A solution for avoiding neck-shoulder pain during computer work may be to implement high intensity voluntary contractions during the computer work. However, it is unknown how this may influence productivity, rate of perceived exertion (RPE) as well as activity and rest of neck-shoulder muscles during computer work. The aim of this study was to investigate short-term effects of a high intensity contraction on productivity, RPE and upper trapezius activity and rest during computer work and a subsequent pause from computer work.

**Methods:**

18 female computer workers performed 2 sessions of 15 min standardized computer mouse work preceded by 1 min pause with and without prior high intensity contraction of shoulder elevation. RPE was reported, productivity (drawings per min) measured, and bipolar surface electromyography (EMG) recorded from the dominant upper trapezius during pauses and sessions of computer work. Repeated measure ANOVA with Bonferroni corrected post-hoc tests was applied for the statistical analyses.

**Results:**

The main findings were that a high intensity shoulder elevation did not modify RPE, productivity or EMG activity of the upper trapezius during the subsequent pause and computer work. However, the high intensity contraction reduced the relative rest time of the uppermost (clavicular) trapezius part during the subsequent pause from computer work (p < 0.04).

**Conclusion:**

Since a preceding high intensity shoulder elevation did not impose a negative impact on perceived effort, productivity or upper trapezius activity during computer work, implementation of high intensity contraction during computer work to prevent neck-shoulder pain may be possible without affecting the working routines. However, the unexpected reduction in clavicular trapezius rest during a pause with preceding high intensity contraction requires further investigation before high intensity shoulder elevations can be recommended as an integrated part of computer work.

## Background

The last decades, the fraction of the working population with primarily sedentary office work performing low load repetitive movement have escalated [1]. In the same period, increases in prevalence of neck-shoulder pain has occurred [2]. Correspondingly, one of the occupations with highest risk for neck-shoulder pain is computer workers [3], and extended computer work is generally considered a precursor for neck-shoulder pain often located in the upper part of the shoulder girdle [4-8]. The high prevalence of neck-shoulder pain in computer workers [3] is commonly considered to be mediated by insufficient upper trapezius rest, causing overload and local circulatory disturbances [9,10].

The individual health problems and economical burden of the escalating prevalence of neck-shoulder pain [11] have stimulated a variety of preventive initiatives. Observations of enhanced occurrence of neck-shoulder pain in computer workers with poor workstation designs and work posture, e.g. keyboard and mouse position, lack of armrests [12] have encouraged extensive ergonomic investigations [13]. The aim for most investigations has been to reduce activity level or interrupt sustained trapezius activity during computer work [5,6]. However, improvements of work posture and workstation designs have not been effective for preventing neck-shoulder pain in computer workers [14].

Several studies have evaluated effects of passive pauses during computer work [15-18]. However, passive pauses are not shown effective for preventing neck-shoulder pain [14] even though a decrease in pain intensity has been reported among workers with chronic neck-shoulder pain [19]. This finding may be explained by the persistent activity of the upper trapezius muscle during both short and long passive pauses during computer work [20]. Crenshaw and colleagues investigated effects of active pauses with voluntary sub-maximal contractions of forearm muscles during computer work [21]. However, the voluntary contractions only provided positive trends on local circulation and muscle activity of forearm muscles during the pauses [21].

In a recent study, we observed a more variable trapezius activity from preceding sub-maximal voluntary contractions (MVC) during computer work, but no effects on average upper trapezius activity and rest [22]. However, because the degree of blood flow following a voluntary contraction is proportional to the intensity of the contraction [23], high intensity voluntary contractions may be necessary for removing metabolites that could accumulate and excite muscle nociceptors [24].

Moreover, strength enhancing physical activity of neck muscles is in a recent review recommended for preventing neck-shoulder pain [3]. Strength training has been shown effective for both preventing and reducing neck-shoulder pain in computer workers [25,26]. This may be due to high intensity contractions are required for gaining strength [27] and attaining high blood flow subsequent to the contractions [23]. However, strength training sessions interrupts daily working routines, and is therefore difficult to put into practice among computer workers [25]. One approach to overcome this obstacle is to implement high intensity strengthening voluntary contractions during computer work. Because only one session with a few high intensity contractions may enhance strength [28], a few implemented high intensity contractions per day may be effective for promoting strength and preventing neck-shoulder pain in computer workers. However, plausible negative effects on productivity, perceived effort, and trapezius activity and rest need to be investigated before high intensity strengthening contractions can be implemented during computer work. Therefore, the aim of this study was to investigate short-term effects of a high intensity voluntary contraction on productivity, rate of perceived effort (RPE) and upper trapezius activity and rest during computer work and a subsequent pause from computer work. The corresponding main hypothesis of the present study was that a MVC does not decrease perceived effort, productivity or upper trapezius activity and rest during subsequent computer work.

## Methods

### Subjects

Eighteen female computer workers from a public work-place in Copenhagen, Denmark with mean (standard deviation) age 41.8 (7.8) years, height 1.70 (0.07) m, weight 74.4 (14.9) kg, and body mass index 26.1 (6.0) participated in the study. The criteria for inclusion were daily computer work and right hand dominance. The exclusion criteria for participation in the study were self-reported excessive blood pressure, angina pectoris, previous disc-prolaps or use of heart or lung medicine. The subjects were informed by the general aims of the study and gave written consent to participate in the study. The experiment was approved by the Local Ethics Committee (KF 01-138/04) and was conducted in accordance with the Helsinki declaration.

### Procedures

The subjects were seated on a height-adjustable chair at a table with appropriate arm support. The computer workstation was adjusted for each subject. The chair was placed upon a solid plate. Two force cells (P.M.H. Elektronik, Copenhagen, Denmark) were attached to the plate on each side of the chair. The handles connected to the force cells were adjusted for each subject, enabling bilateral isometric MVC of shoulder elevation with straight arms and wrists in a neutral position. The instruction to the subjects during the MVC was to enhance the force to a maximal level in a controlled manner, hold the maximal force level for 4–5 s, and then reduce the force in a controlled manner.

The standardized computer mouse work was similar to previously described [29], consisting of a computer mouse working task using a digitizer. The task consisted of pointing a cursor to targets becoming activated by clicking the mouse button. Lines were automatically drawn between consecutively activated targets, and the task was to activate targets in a specific order to duplicate a drawing shown in the upper right corner of the computer screen (pixel resolution: 0.3 mm. screen resolution: 1024 x 768 pixels). As soon as the drawing was correctly completed, new targets appeared. The subject was instructed to work at maximal pace and make as many drawings as possible. The number of correct drawings completed was calculated for each session of computer work.

First, the subjects performed ~15 min of the standardized computer mouse work to get familiar with the procedure. Then, 2 sessions of computer mouse work were performed. Each session lasted 15 min and started and ended with 1 min pause. The order of the next two sessions of computer work was randomized between the subjects. One session started with a MVC of shoulder elevation followed by 1 min pause and 15 min of computer mouse work. The other session of computer mouse work was identical, but without a preceding MVC. After performing the three sessions of computer mouse work, for normalization of the electromyographical (EMG) signal (see below) the subjects performed at least two additional MVCs of shoulder elevation with 1 min pause between the contractions.

### Electromyography

During the entire experiment, surface EMG signals were recorded from the dominant upper trapezius muscle with bipolar surface electrodes (Ag-AgC1, type 72501-K, Ambu, Denmark) using a datalogger (Logger Teknologi HB, Lund, Sweden). Therefore, bipolar EMG electrodes (10 mm inter-electrode distance) were placed ~20% lateral to the midpoint between the origin and insertion of the uppermost muscle fibers of the trapezius (clavicular part), and ~20% medial to the midpoint between the acromion and the C7 vertebra (descending part). The reference electrode (Ag-AgCl, type 72001-K, Ambu, Denmark) was placed on the acromion. The EMG signals were amplified (Datalogger, Logger teknologi HB, Sweden), AD converted at 2 kHz and recorded on computer via a laboratory interface (CED 1401, Spike2 software, Cambridge Electronic Devices, UK). The signals were analogue band-pass filtered (10–400 Hz, 8^th ^order, Butterworth). The signals were visually verified and high-pass filtered (cut-off 10 Hz), full wave rectified and root-mean-square (RMS) converted with windows of 100 ms duration throughout all recordings of the study.

For normalization of the EMG signal during computer work, the maximal EMG amplitude (EMG_max_) was calculated as the highest RMS amplitude during MVC obtained with a moving window (1-s duration and 100 ms steps) [30]. The resting EMG signal was calculated from 1 min of instructed rest with closed eyes and complete arm support. The time period of 5 s with lowest RMS value during the instructed rest was quadratically subtracted from all other EMG signals [20]. The average RMS from the 1 min pause and the first 5 min and last 5 min of each computer mouse sessions were calculated. In the same time-periods, the relative rest time (RRT) being the total duration of EMG activity < 0.5% EMG_max _using a time resolution of 0.2 s was calculated [31].

### Rate of perceived exertion

The participants were asked about the rate of perceived exertion (RPE) using Borg's CR 10-scale (Borg 1990) of the dominant neck-shoulder region during the end of each pause and session of computer mouse work

### Statistics

Muscle parts (clavicular, descending), conditioning contraction (MVC or rest), and time (first or last 5 min of the computer work session) were introduced as factors in a repeated measure ANOVA for EMG amplitude and RRT during computer work as dependent variables. To analyse effects of conditioning contraction on the 1 min pause from computer work, a repeated measure ANOVA with intra-muscular parts and conditioning contraction as factors for EMG amplitude and RRT as dependent variables were performed. Effects of conditioning contraction on the RPE during subsequent pause and computer work were tested with a repeated measure ANOVA with conditioning contraction and condition (pause or computer work) as factors. Effects of a conditioning contraction on productivity during computer work were tested with a repeated measure ANOVA with conditioning contraction and time as factors. If the assumption of sphericity was not met, a Greenhouse Geisser correction was applied. Bonferroni corrected post-hoc tests were performed to locate differences. The level of significance was set to p < 0.05. Mean +/- SD are reported through the text.

## Results

### Rate of perceived effort (RPE)

**Figure 1:**
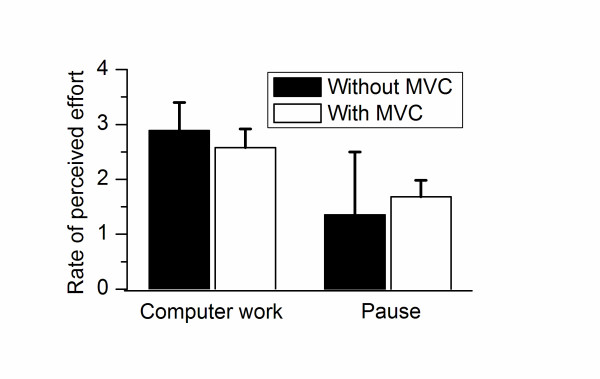
**Average (+/- SE) rate of perceived effort in the last 5 min of computer mouse work and in the end (last 5 s) of the 1 min pause with and without preceding maximal voluntary shoulder elevation**.

A significant reduction in RPE was found in the pauses compared to computer mouse work (Figure 1). However, no significant effect of pause type was found for RPE (p = 0.56).

### Trapezius activity and rest during pauses from computer mouse work

**Figure 2:**
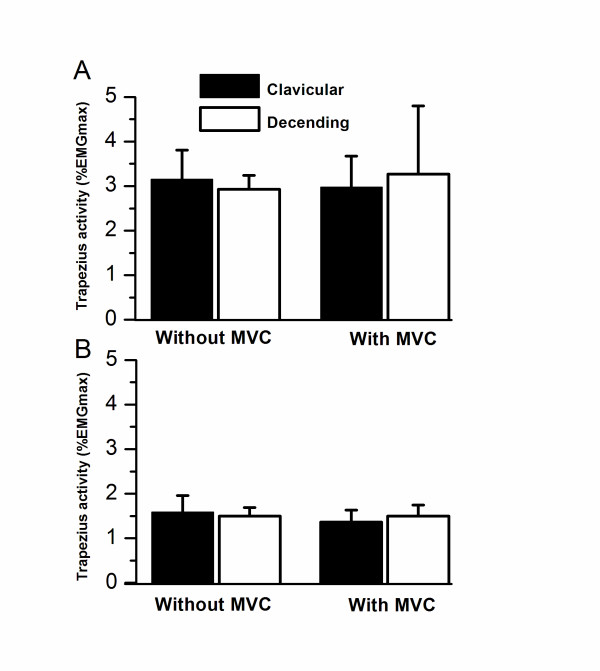
**Average (+/- SE) EMG activity (RMS % EMG_max_) from the clavicular and descending part of the trapezius during A) computer mouse work and B) 1 min pause with and without preceding maximal voluntary shoulder elevation**.

**Figure 3:**
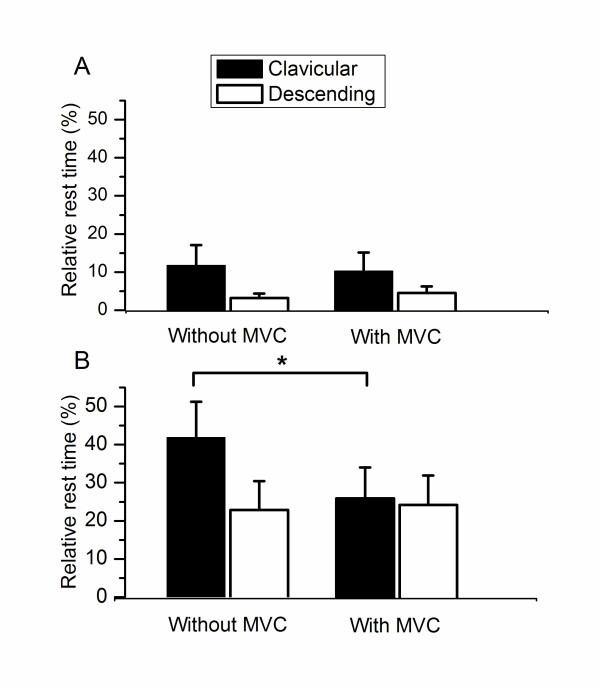
**Average (+/- SE) relative rest time (%) from the clavicular and descending part of the trapezius during A) computer mouse work and B) 1 min pause with and without preceding maximal voluntary shoulder elevation**. * p < 0.04.

During the 1 min pause after a session of computer work, an average EMG activity of 1.6 +/- 1.6%EMG_max _in the clavicular, and 1.5 +/- 0.8%EMG_max _in the descending part of the trapezius was observed (Figure 2B). The average RRT during the 1 min pause subsequent to a session of computer work was 42.0 +/- 39.9% in the clavicular, and 26.4 +/- 34.5% in the descending part. A significant interaction between muscle parts and pause type was observed for RRT (p < 0.04), in which a lowered RRT of the clavicular part subsequent to a MVC was observed, while no significant changes were observed for the descending part (Figure 3B).

### Trapezius activity and rest during computer mouse work

The EMG activity of the clavicular and descending parts of the trapezius during computer work is illustrated in Figure 2A. The average EMG activity during the first 5 min of computer work without preceding MVC was 3.0 +/- 2.5%EMG_max _in the clavicular, and 3.3 +/- 1.5%EMG_max _in the descending part of the trapezius (Figure 2A). The average RRT during the first 5 min of computer work without preceding MVC was 11.7 +/- 23.0% in the clavicular, and 3.2 +/- 4.9% in the descending part of the trapezius (Figure 3A). No interactions were observed for EMG activity and RRT for intra-muscular part (p = 0.33), pause type (p = 0.32) or time during computer work (p = 0.34).

### Productivity during computer work

During the first 5 min of computer work without preceding pause with MVC, the subjects finished on average 51.3 +/- 6.3 drawings. During the first 5 min of computer work preceding pause with MVC, the subjects finished on average 50.3 +/- 6.0 drawings. In the last 5 min of computer work without preceding pause with MVC, the subjects finished on average 49.1 +/- 7.3. In the last 5 min of computer work with preceding pause with MVC, the subjects finished on average 49.1 +/- 5.3. No significant effect was found for pause type (p = 0.52), but the subjects finished less drawings in the last 5 compared with the first 5 min of computer work (p < 0.01).

## Discussion

The main finding of the present study was that a high intensity shoulder elevation contraction did not modify RPE, productivity or EMG activity of the upper trapezius during the subsequent pause and computer work. However, the high intensity shoulder elevation reduced the relative rest time of the uppermost (clavicular) trapezius part during the subsequent pause from computer work. The suitability for implementing MVCs during computer work for preventing neck-shoulder pain in computer users will be discussed in the following.

An average activity of the upper trapezius of ~3% EMG_max _was observed during the computer mouse work. A similar level of sustained trapezius activity has previously been observed with the same type of computer task [20,32-34]. Moreover, the observed low RRT of the upper trapezius during computer work confirms the low level of rest of the upper trapezius during computer work in office workers [22]. Comparable low levels of sustained trapezius activity and RRT have previously been documented as a precursor for development of neck-shoulder pain [35].

The implementation of passive pauses during computer work is a frequently recommended preventive strategy for attaining sufficient periods of rest of the upper trapezius muscle [17,36,37]. However, even quite long pauses (4 min) from computer work are insufficient for attaining complete rest of the upper trapezius muscle [20]. This finding is supported by the observation in the present study of an average upper trapezius activity of 1.5% EMG_max_, and a relatively low RRT (42% in clavicular and 26% in descending) trapezius during the 1 min pause from computer work. Consequently, the present study supports that passive pauses from computer work may not be sufficient for the prevention of neck-shoulder pain in computer workers [14].

A suggested preventive strategy for neck-shoulder pain in computer workers is implementation of active pauses during computer work [15,21,36]. However, no significantly improved conditions of muscle activity, local circulation or discomfort have been documented from different types of active pauses during computer work, i.e. stretching exercises [15,36], walking [16], sub-maximal contractions [21] and gymnastic exercises [37].

One reasoning for investigating implementation of a MVC of shoulder elevation during computer work is that intermittent voluntary contractions of high intensity are well known to subsequently enhance local muscle blood flow [23]. A single MVC of shoulder elevation may therefore reduce trapezius activity during subsequent computer work by washing out accumulated metabolites [24]. A recent study found a beneficial long term effect on neck-shoulder pain from strength training within female computer workers, but also an acute increase in pain after the training session [26]. By implementing high intensity strengthening voluntary contractions in short breaks within the actual work, it may be easier for the workers to find time to perform the strength training. However, a draw back of implementing high intensity voluntary contractions into computer work could be some muscle fatigue and discomfort that could interfere with work productivity, perceived effort and trapezius activity and rest.

Therefore, the main hypothesis of the present study was that a MVC does not influence perceived effort, productivity or upper trapezius activity and rest during subsequent computer work. In accordance with the hypothesis, a preceding MVC did not affect perceived effort, productivity or upper trapezius activity (no increase considered a sign of muscle fatigue was found) and rest during computer work. Therefore, implementation of MVC in the work itself may be a well-suited alternative to strengthen the neck-shoulder muscles, and thereby potentially prevent neck-shoulder pain in computer workers.

However, a MVC was surprisingly followed by a reduced rest of the uppermost (clavicular) trapezius part during the subsequent pause. Lack of rest in the upper trapezius during computer work is considered a plausible cause to the high prevalence of neck-shoulder pain among computer workers [6]. The reduced rest of the uppermost trapezius during the subsequent pause indicates an enhanced excitability of the trapezius motoneurons. However, this physiological explanation needs to be further investigated. Therefore, the reduced upper trapezius rest during a pause from computer work with preceding MVC requires further investigation before high intensity shoulder elevations can be recommended as an integrated part of computer work.

The observation of no significant negative effects from an implemented high intensity contraction may be caused by a methodological limitation of the study. However, the observation of a significant effect of a high intensity contraction on relative rest time of the clavicular trapezius indicates a sufficiently high reliability for detecting differences in at least relative rest time from a high intensity contraction. Moreover, the experimental setup and applied EMG variables are well-established and validated in several studies [20,32,35], supporting that no type II error has occurred in this study.

## Conclusion

Work-site strength training sessions are previously shown to prevent and reduce neck-shoulder pain in computer workers, but difficult to withstand for longer periods of time. This study investigated plausible effects of implementing a high intensity voluntary contraction into computer work. However, before high intensity voluntary contractions can be recommended as an implemented part of computer work, plausible negative effects on productivity, perceived effort and trapezius activity and rest need to be investigated. No such negative effects during computer work were observed. Therefore, implementation of high intensity voluntary contractions during computer work may be a well-suited approach for preventing neck-shoulder pain in computer workers. However, reduced upper trapezius rest during a pause from computer work with preceding MVC requires further investigation before high intensity shoulder elevations can be recommended as an integrated part of computer work.

## Competing interests

The authors declare that they have no competing interests.

## Authors' contributions

MKL coordinated and performed the study. MKL and HBO analyzed the data. MKL and AH made the statistical analyses and wrote the first draft of the manuscript. All authors contributed to the conception, design, interpretation of data, and critically revising the manuscript. All authors approved the final manuscript.

## Pre-publication history

The pre-publication history for this paper can be accessed here:

http://www.biomedcentral.com/1471-2474/10/101/prepub
